# Familial Hypercholesterolemia Patients with COVID-19—Effective Cholesterol-Lowering Therapy is Urgent both during and after Infection

**DOI:** 10.31083/j.rcm2312410

**Published:** 2022-12-19

**Authors:** Alpo Vuorio, Frederick Raal, Petri T. Kovanen

**Affiliations:** ^1^Mehiläinen Airport Health Centre, 01530 Vantaa, Finland; ^2^Department of Forensic Medicine, University of Helsinki, 00100 Helsinki, Finland; ^3^Faculty of Health Sciences, University of Witwatersrand, 2193 Johannesburg, South Africa; ^4^Wihuri Research Institute, 00290 Helsinki, Finland

**Keywords:** familial hypercholesterolemia, statins, PCSK9 inhibitors, endothelial dysfunction, atherosclerotic cardiovascular disease, COVID-19

## Abstract

Heterozygous familial hypercholesterolemia (HeFH) patients are the prime example of subjects who are at high risk for both acute myocardial infarction (AMI) and ischemic stroke during, and post, SARS-CoV-2 infection. HeFH *per se*, if left untreated, results in premature clinical atherosclerosis often presenting in the fourth or fifth decade of life. The other concern in HeFH is endothelial dysfunction which is already evident from early childhood. In untreated HeFH patients, the severe hypercholesterolemia causes endothelial dysfunction from an early age, and as a result thereof, atherosclerotic lesions develop prematurely, particularly in the coronary arteries, and result in further endothelial dysfunction and inflammation in these critical segments of the arterial tree. As the pre-existing endothelial dysfunction in HeFH patients is most likely sensitive to further direct and indirect SARS-CoV-2 virus-dependent damage, we can infer that HeFH serves as an example of a comorbidity that predicts a poorer prognosis with COVID-19 infection. Indeed, a large US national database study showed that patients diagnosed with HeFH and SARS-CoV-2 infection had significantly increased Annualized Incidence Density Rates (AIDRs) of AMI when compared to matched HeFH controls not having been diagnosed with SARS-CoV-2 infection. Effective cholesterol lowering is essential for the prevention, or at least alleviation, of the detrimental effects of SARS-CoV-2 infection among HeFH patients. Due to the pre-existing subclinical or even clinical atherosclerotic cardiovascular disease in subjects with HeFH, cholesterol-lowering treatment needs to be continued or, better still, intensified during, and for an extended period post, SARS-CoV-2 infection.

## 1. Introduction

In a very large Swedish case-control study, it has been clearly demonstrated that SARS-CoV-2 infection is a risk factor both for acute myocardial infarction (AMI) as well as ischemic stroke [1]. In this study, the incidence rate ratio for AMI was 2.89 (95% CI 1.51–5.55) for the first week, 2.53 (1.29–4.94) for the second week, and 1.60 (0.84–3.04) for weeks 3 and 4 post COVID-19 infection. The respective incidence rate ratios for ischemic stroke were 2.97 (1.71–5.15) in the first week, 2.80 (1.60–4.88) in the second week, and 2.10 (1.33–3.32) in weeks 3 and 4 following COVID-19. Additionally, the American retrospective analysis of COVID-19 patients with a diagnosis of acute AMI registered in the National COVID Cohort Database revealed that COVID-19 patients with AMI undergoing early invasive coronary angiography had a worse prognosis compared to those without COVID-19 [2]. Impressively, in this study, nearly 80% of COVID-19 positive and negative groups with AMI had hypercholesterolemia. Much of the increase in risk and worse prognosis following AMI or stroke can be explained by the fact that COVID-19 is largely an endothelial disease [3, 4]. Furthermore, it has been recently shown that patients with COVID-19 not only have an acute impairment of endothelial function but that the endothelial dysfunction persists for up to 6 months, or even longer, post-infection [5]. This period potentially increases the residual risk, especially for atherosclerotic complications among patients who are at high risk for a pre-existing atherosclerotic cardiovascular disease (ASCVD) and those who already have ASCVD.

Heterozygous familial hypercholesterolemia (HeFH) patients are the prime example of subjects who are at high risk for both AMI and ischemic stroke during and after COVID-19 [6]. HeFH *per se*, if left untreated, results in premature clinical atherosclerosis often presenting in the fourth or fifth decade of life as a result of the markedly elevated lifelong burden of elevated serum low-density lipoprotein cholesterol (LDL-C) [7, 8, 9]. The other concern in HeFH is endothelial dysfunction that manifests itself from early childhood. The endothelium is under stress since in these patients, besides an increased serum LDL-cholesterol level, the level of serum lipoprotein(a) [Lp(a)] is also frequently increased, and both tend to disrupt normal endothelial function [10]. In this narrative review, we highlight the current understanding of COVID-19-related AMI and ischemic stroke among HeFH patients. In addition, we discuss the preventive impact of lipid-modifying agents, especially of statins and proprotein convertase subtilisin/kexin type 9 (PCSK9) inhibitors in SARS-CoV-2 infection, not only in HeFH patients but also those with the extremely rare and much more severe form of FH, the homozygous form of FH (HoFH), a condition in which AMI can occur in the first or second decade of life [11]. Finally, we shed light on the challenges posed by potentially accelerated atherosclerosis among HeFH patients with previous SARS-CoV-2 infection.

## 2. COVID-19 and Risk for AMI

A large US national database study of over 301,628,074 individuals showed that patients diagnosed with diagnosed or probable HeFH and SARS-CoV-2 infection had significantly increased Annualized Incidence Density Rates (AIDRs) of AMI when compared to matched HeFH controls not having been diagnosed with SARS-CoV-2 infection [12]. Particularly high AIDR was shown among the diagnosed FH patients who had an established ASCVD. In this group, the AIDR of AMI incidence was 11.3% when compared with 2.3% (*p *< 0.0002) in HeFH patients with ASCVD but without COVID-19 [12]. The AIDR was lower among diagnosed HeFH patients who did not have a diagnosed ASCVD when compared with those who had a diagnosed ASCVD. AIDRs of AMI among the diagnosed HeFH patients with and without COVID-19 were 3.2% and 0.30 %, respectively (*p <* 0.0002).

Interestingly, but counterintuitively, the relative increase of AMI among diagnosed HeFH patients with COVID-19, when compared with diagnosed HeFH patients with pre-existing ASCVD and COVID-19, was higher in those without ASCVD. Thus, in the group of HeFH with COVID-19 but no pre-existing ASCVD, the AIDR of AMI was increased about 10-fold, whereas in the group of HeFH with COVID-19 and pre-existing ASCVD the respective increase was about 5-fold. A likely explanation is that statins may have been used more frequently and intensively in the group of HeFH with clinically diagnosed ASCVD and that the mitigating effect of statins on the SARS-CoV-2 infection was therefore more evident in this patient group [6]. Altogether, the large study by Myers and coworkers [12] demonstrated that in HeFH patients with SARS-CoV-2 infection and pre-existing ASCVD, the proportional increase in risk for AMI was only about half of that in the HeFH group without clinically diagnosed pre-existing ASCVD. This result confirms the notion that also the subclinical atherosclerotic lesions in the coronary arteries of non-symptomatic HeFH patients are potentially highly vulnerable to SARS-CoV-2 infection and the associated cytokine storm. Accordingly, when predicting absolute numbers of COVID-19 in HeFH, it can be assumed that the AIDR of AMI in the HeFH patients with or without ASCVD and who need to be hospitalized for severe COVID-19 would be even higher than that observed in the non-hospitalized and hospitalized population-based study discussed above.

## 3. COVID-19 and Ischemic Stroke

Our knowledge related to COVID-19 and the risk of ischemic stroke is mainly based on studies carried out in the general population [13, 14, 15]. In a Spanish prospective multicenter cohort study of 701 patients (mean age 72.3 ± 13.3 years, 60.5% men), the COVID-19 patients with ischemic stroke had more severe strokes and significantly higher mortality than those without SARS-CoV-2 infection [13]. The authors of this study emphasized that future studies need to be conducted to determine whether the subtype of ischemic stroke with COVID-19 has a different pathophysiology compared to ischemic stroke without SARS-CoV-2 infection. On the other hand, in another recent study based on the data obtained from the Swiss Stroke database of 2341 stroke patients, it was observed that COVID-19 patients with stroke tended to have 3-month functional outcomes comparable to those stroke patients without SARS-CoV-2 infection [15]. In this study, about 64% of ischemic stroke patients had hypercholesterolemia but only about 50% were receiving cholesterol-lowering medication. These percentages led the authors to speculate that COVID-19 may be a trigger of stroke, especially among patients having a high load of traditional cerebrovascular risk factors, a conclusion which was recently confirmed in 10,881 hospitalized Filipino COVID-19 patients with stroke [16]. Moreover, the observation that ischemic stroke with COVID-19 usually occurs in subjects with other cardiovascular risk factors has been demonstrated in a very large registry study (N = 27,676) comprising several ethnicities [14].

Traditionally, HeFH patients have been considered to fall into the category of patients with an elevated risk of ischemic stroke, particularly those not receiving statin treatment. However, this notion was based on the results of a study carried out before the statin era [17]. In the statin era, the risk of ischemic stroke among HeFH patients has reduced markedly, and among the patients treated with statins, the risk is similar to that in the background population without HeFH [18]. Unfortunately, most HeFH patients remain undiagnosed, and, even if diagnosed, they are either not taking statins or their statin dose is sub-optimal [19, 20]. In light of the foregoing, we argue that untreated HeFH patients with COVID-19 have an elevated risk of a more serious ischemic stroke [21]. To test this hypothesis, however, a large multicenter and multi-ethnic registry study is required.

## 4. Lipid-Lowering Drugs and COVID-19

Several ongoing randomized controlled trials on the effects of statin treatment during COVID-19 are ongoing [22]. Our current knowledge of the clinical benefits of statin treatment of COVID-19 patients is based predominantly on retrospective analyses and systematic reviews [23, 24, 25, 26, 27, 28, 29, 30]. The proposed beneficial mechanisms of action of statins include (1) inhibition of the viral RNA-dependent RNA polymerase and the main protease, (2) immunomodulatory and anti-inflammatory effects, (3) antithrombotic effects, and (4) improvement of endothelial dysfunction [6, 26, 28, 31, 32]. Furthermore, because of the PCSK9–dependent dampening of antiviral cellular responses, the PCSK9 inhibitors could also improve the prognosis of SARS-CoV-2 infection [33]. In addition, PCSK9 inhibitors decrease serum Lp(a) concentration, albeit modestly, which in turn could further lessen the magnitude of endothelial dysfunction [33].

Effective cholesterol lowering is essential for the prevention, or at least alleviation, of the detrimental effects caused by SARS-CoV-2 infection in HeFH patients [6, 34]. In HeFH patients without cholesterol-lowering medication, the severe hypercholesterolemia causes endothelial dysfunction from an early age onwards, early development of atherosclerotic lesions, particularly in the coronary arteries, and, therefore, SARS-CoV-2 infection at any age is likely to result in further endothelial dysfunction and inflammation in coronary arteries and other critical segments of the arterial tree [6]. As the pre-existing severe endothelial dysfunction in HeFH patients is most probably sensitive to the additional direct and indirect virus-dependent endothelial damages, we can infer that HeFH serves as an example of a comorbidity that predicts a poorer prognosis with COVID-19 infection [35].

Due to the pre-existing subclinical, or even clinically overt ASCVD in subjects with HeFH, cholesterol-lowering treatment needs to be continued or, preferably even intensified, during the initial illness period following SARS-CoV-2 infection and for an extended period after acute COVID-19 [6, 36]. This applies especially to statins, the use of which has been associated with improved prognosis in most reported registry studies among hospitalized COVID-19 patients [37, 38, 39]. Currently, several randomized controlled studies on the use of statins in COVID-19 patients are in progress and the favorable effect of statins will probably be confirmed in these studies [22]. The beneficial effects of statins most likely result from their anti-inflammatory and antithrombotic effects, both as a result of their LDL-cholesterol-lowering ability and their putative beneficial pleiotropic effects [31, 32]. Interestingly, a recent study demonstrated that the SARS-CoV-2 main protease ($M^{\text{pro}}$) adversely affects microvascular endothelial cells in the brain [40]. It has been found, at least *in silico*, that statins can directly interact with the SARS-CoV-2 main protease $M^{\text{pro}}$, fluvastatin being one such example [41]. However, the findings from this laboratory-based *in vitro* study needs to be confirmed in clinical trials. In addition to statins, PCSK9 inhibitors and fenofibrate may improve the prognosis of COVID-19 when continued during the SARS-CoV-2 infection [33, 42].

Paxlovid is a new antiviral medication recommended for use in subjects at high risk of severe SARS-CoV-2 infection, such as patients with HeFH [43]. Unfortunately, the simultaneous use of Paxlovid and statin may result in serious interactions [44]. While it is essential to continue statin treatment during the 5-day Paxlovid treatment, simvastatin and lovastatin need to be substituted with another statin [45, 46]. The substitution can be done with either pravastatin or fluvastatin. If the patient is on atorvastatin or rosuvastatin, a reduction of the dose is recommended if Paxlovid is prescribed.

## 5. Special Features of Cholesterol Lowering in Different FH Patient Groups with COVID-19 

In general, it appears that HeFH patients have been treated less effectively since the COVID-19 lockdown. This is supported by a retrospective telephone study of 260 HeFH patients from a lipid clinic which demonstrated that the number of lipid laboratory tests and cardiology consultations declined following a COVID-19 lockdown in Italy [47]. As far as we are aware, there is only one case report of a HeFH patient with SARS-CoV-2 infection [48]. In this study, the HeFH patient with COVID-19 also had hypertrophic cardiomyopathy and suffered sudden cardiac death. Marziliano *et al*. [48] remind us of the importance of autopsies and post-mortem studies which may enlighten us further about the pathogenic mechanisms related to SARS-CoV-2 infections.

Next, we wish to pay particular attention to decisions regarding cholesterol-lowering treatment that need to be considered in special HeFH patient groups during a SARS-CoV-2 infection. In particular, we discuss pregnant HeFH and HoFH mothers, HeFH and HoFH children, and older HeFH patients.

### 5.1 Pregnant HeFH Mothers with COVID-19

During pregnancy, serum LDL-C increases by about 30%, and serum triglyceride (TG) concentrations may even double [49]. Serum lipoprotein(a) [Lp(a)] levels also increase [50]. In women with HeFH, these pregnancy-dependent changes are even more significant, since serum LDL-C and often also Lp(a) are elevated already since birth [6]. Increased serum LDL-C, Lp(a), and TG, individually, and particularly in combination, aggravate vascular endothelial dysfunction. Statins are not recommended for use during pregnancy, whereas the use of bile acid sequestrants and/or LDL apheresis is possible [42, 43, 51, 52]. Recently, however, the FDA has removed the contraindication for statins in pregnant women with established ASCVD or in those at very high risk for ASCVD, such as women with HoFH [53]. The risk factors predisposing to pregnancy complications in mothers infected with SARS-CoV-2 are not well described or well-known [54]. The decision to use statins during pregnancy in HeFH mothers with COVID-19 requires an individualized approach. It can be assumed that, in most cases, the benefit of statin use in a pregnant HeFH woman with SARS-CoV-2 infection is justified as the benefit likely outweighs any potential harm [55]. Pravastatin can be preferentially recommended because it has been shown not to alter fetal cholesterol metabolism [56].

### 5.2 HeFH and HoFH Children with COVID-19

In general children with SARS-CoV-2 infection have a low hospitalization rate [57]. However, concern regarding post-COVID-19 complications or conditions has been raised [58]. In a large 90-day follow-up cohort study following SARS-CoV-2 infection among 1884 children (median age 3 years) 9.8% of hospitalized children suffered from post-COVID-19 complications or conditions [59], pre-existing chronic illnesses being risk factors for the likelihood of developing a post-COVID-19 condition [60].

Whilst there are no published data regarding post-COVID-19 conditions in children with FH, we want to draw special attention to the features of their treatment during SARS-CoV-2 infection. Most importantly, children hospitalized for COVID-19 infection having HeFH, or the more severe homozygous form of FH (HoFH), need to continue statin therapy. In HoFH children, the onset of atherosclerosis can occur already *in utero*, and in these children, if not intensively treated with LDL-cholesterol-lowering pharmacotherapy in combination with mechanical removal of plasma LDL particles (LDL apheresis), very early AMI is common, often in the first or second decade of childhood [11, 61]. Thus, to prevent the progression of accelerated atherosclerosis to clinical manifestations, LDL apheresis is essential for patients with severe HoFH who have not responded adequately to the available cholesterol-lowering pharmacotherapies [62]. Furthermore, it has been reported that regular LDL apheresis in HoFH patients was hampered during the COVID-19 pandemic because the apheresis unit had other liabilities, or because patients were reluctant to come to the hospital during the pandemic [34, 63]. LDL apheresis helps to improve endothelial dysfunction in HoFH patients which is of utmost importance during SARS-CoV-2 infection, since the viral infection and the associated hyperinflammatory responses further impair the anti-atherosclerotic and antithrombotic functions of the endothelium, as discussed above [3, 64, 65]. In addition to the acute harmful effect, discontinuation of LDL apheresis may also have long-term adverse cardiovascular consequences because the LDL-C levels will increase.

### 5.3 Older HeFH Patients with COVID-19

The United Nations (UN) has expressed concern that aging populations are especially vulnerable to COVID-19 infection [66]. On the other hand, older patients with COVID-19 are not a homogenous group of patients, and health and safety measures need to be tailored according to individual needs [67]. In a large population-based study, age was a risk factor for COVID-19 causing more deaths among the population aged over 40 years when compared to younger subjects [68]. One explanation might be that circulating chitinase 3-like-1 (CHI3L1) levels increase with age [69]. CHI3L1 is a stimulator of the angiotensin-converting enzyme 2 (ACE2) and viral spike protein priming proteases [69]. The dynamic nature of the different COVID-19 waves is illustrated by the fact that among hospitalized patients in Italy and the UK the mortality of elderly people aged 65 or over was significantly lower during the first wave of COVID-19 (February–June 2020) than during the second wave (October 2020–March 2021) [70]. The explanation provided by Verduri and co-authors [70] was that less frailty elderly were hospitalized during the second wave of COVID-19.

In general, older HeFH patients are at risk for COVID-19 because of their greater cumulative cholesterol burden and advanced ASCVD [71, 72]. Cumulative cholesterol burden can be measured using the formula LDL-C concentration (mmol/L) × age (years) [73]. Accordingly, the cholesterol burden of a 70-year-old untreated FH patient with an LDL-C level of 5 mmol/L is 350, while that of a 70-year-old non-FH person with an LDL-C level of 3 mmol/L is only 210. Moreover, at the age of 70, an HeFH patient who has taken statins from the age of 30 and who has reached an LDL-C level of 2.5 mmol/L will have a cholesterol burden of 250. The significant increase in the burden, particularly in untreated older FH patients, would then translate into a worse cardiovascular prognosis. The pre-existing highly increased cardiovascular risk is one of the reasons why older HeFH patients have a very high risk of cardiac events and complications during COVID-19 [74]. The clinician, therefore, needs to consider not only the continuation of the current lipid-lowering treatment but even the intensification of the therapy during and after COVID-19 infection [6, 37, 74].

## 6. Future Challenges

The COVID-19 pandemic has impacted the management of FH patients with either the heterozygous or the homozygous form of the disease, particularly in middle- and low-income countries [75]. For example, in Brazil, there was a total closure of a major lipid clinic at the Heart Institute at the University of Paulo School Hospital during the first wave of the pandemic. All attempts to continue cholesterol-lowering therapy among FH patients are essential. However, it is likely that some FH patients who were previously attending lipid clinics and who were receiving statin therapy stopped attending clinics, or were lost to follow-up, during the pandemic [47].

An analysis of very large healthcare databases from the US Department of Veterans Affairs has demonstrated that the time of increased cardiovascular risk, even for non-hospitalized COVID-19 patients, may last up to one year after the infection [76]. The increased cardiovascular risk applies to cardiac dysrhythmias, ischemic and non-ischemic heart disease, pericarditis, myocarditis, heart failure, cerebrovascular disorders, as well as to thromboembolic disease. In this context, ongoing effective cholesterol-lowering treatment or the introduction of cholesterol-lowering therapy, particularly in patients with FH, is paramount [77]. As many HeFH patients are sub-optimally treated, even before SARS-CoV-2 infection, it is critical to ensure that cholesterol-lowering therapy is continued and that statin doses are even increased, or additional lipid-lowering therapies are added, to achieve the currently recommended LDL-cholesterol targets.

A very recent pre-published large cohort study of the databases of the US Department of Veterans Affairs showed that, when compared to a primary SARS-CoV-2 infection, a re-infection increases all-cause of mortality, hospitalization risk, as well as the occurrence of adverse outcomes including cardiovascular disorders [78]. These risks were present in both the acute and post-acute phases of SARS-CoV-2 reinfection. Al-Aly *et al*. [78] concluded that prevention of a second or subsequent SARS-CoV-2 infection must be the goal. The aim to prevent SARS-CoV-2 reinfection certainly applies to FH patients who are at increased risk of SARS-CoV-2 infection-related cardiovascular adverse events.

Finally, since both HeFH and HoFH patients are at high-risk for cardiovascular complications from COVID-19 infection, they should protect themselves from reinfection. A recent study of 95,000 Rhode Island residents showed that reinfection was relatively high among unvaccinated individuals, while vaccination following on COVID-19 recovery reduced reinfection risk by almost 50% [79]. Moreover, a Swedish retrospective registry study revealed that individuals who have recovered from a previous SARS-CoV-2 infection get additional protection against reinfection if they have been vaccinated [80].

## 7. Conclusions

In FH, a high serum LDL-C concentration, often accompanied by a high concentration of serum Lp(a) results in chronic endothelial dysfunction from early childhood [10]. During COVID-19, the associated cytokine storm and infection of endothelial cells by the virus further worsen endothelial dysfunction, whereupon the thrombotic/fibrinolytic balance of the endothelium is altered to promote a procoagulant state and thrombosis in the microvasculature of multiple organs such as the heart and brain [6]. Based on the above data, we can conclude that in FH patients, effective prevention of SARS-CoV-2 infection-related adverse events must include not only adequate vaccination but also the introduction or ongoing use of effective cholesterol-lowering medication.

## Abbreviations

AMI, acute myocardial infarction; AIDR, annualized incidence density rate; ACE2, angiotensin-converting enzyme 2; ASCVD, atherosclerotic cardiovascular disease; CHI3LI, chitinase 3-like-1; FH, familial hypercholesterolemia; HeFH, heterozygous familial hypercholesterolemia; HoFH, homozygous familial hypercholesterolemia; LDL-C, low-density lipoprotein cholesterol; Lp(a), lipoprotein(a).
